# Prosocial deficits in behavioral variant frontotemporal dementia relate to reward network atrophy

**DOI:** 10.1002/brb3.807

**Published:** 2017-09-14

**Authors:** Virginia E. Sturm, David C. Perry, Kristie Wood, Alice Y. Hua, Oscar Alcantar, Samir Datta, Katherine P. Rankin, Howard J. Rosen, Bruce L. Miller, Joel H. Kramer

**Affiliations:** ^1^ Department of Neurology University of California San Francisco CA USA; ^2^ Sandler Neurosciences Center San Francisco CA USA

**Keywords:** empathy, generosity, giving, neurodegenerative, prosocial

## Abstract

**Introduction:**

Empathy and shared feelings of reward motivate individuals to share resources with others when material gain is not at stake. Behavioral variant frontotemporal dementia (bvFTD) is a neurodegenerative disease that affects emotion‐ and reward‐relevant neural systems. Although there is diminished empathy and altered reward processing in bvFTD, how the disease impacts prosocial behavior is less well understood.

**Methods:**

A total of 74 participants (20 bvFTD, 15 Alzheimer's disease [AD], and 39 healthy controls) participated in this study. Inspired by token‐based paradigms from animal studies, we developed a novel task to measure prosocial giving (the “Giving Game”). On each trial of the Giving Game, participants decided how much money to offer to the experimenter, and prosocial giving was the total amount that participants gave to the experimenter when it cost them nothing to give. Voxel‐based morphometry was then used to identify brain regions that were associated with prosocial giving.

**Results:**

Prosocial giving was lower in bvFTD than in healthy controls; prosocial giving in AD did not differ significantly from either of the other groups. Whereas lower prosocial giving was associated with atrophy in the right pulvinar nucleus of the thalamus, greater prosocial giving was associated with atrophy in the left ventral striatum.

**Conclusion:**

These findings suggest that simple acts of generosity deteriorate in bvFTD due to lateralized atrophy in reward‐relevant neural systems that promote shared feelings of positive affect.

## 1 INTRODUCTION

Interpersonal relationships are essential for the survival of humans and other highly social species. Prosocial behaviors—behaviors that prioritize the needs of others over one's own—strengthen kinship bonds, maintain group cohesiveness, promote interpersonal safety, and facilitate resource‐sharing (de Waal, 2012; de Waal & Suchak, 2010; Decety, 2011). In non‐human animals, prosociality is assessed by measuring other‐focused affiliative actions including consolation, targeted helping, cooperation, and giving (Boesch, 1994; de Waal, Leimgruber, & Greenberg, 2008; Fraser & Bugnyar, 2010; Kuczaj et al., 2015; Palagi, Paoli, & Tarli, 2004; Plotnik, Lair, Suphachoksahakun, & de Waal, 2011; Warneken, Hare, Melis, Hanus, & Tomasello, 2007). Elephants and chimpanzees use physical contact to console kin who are injured or ill, and some primates and rats give food to conspecifics when they could choose to feed only themselves (Ben‐Ami Bartal, Decety, & Mason, 2011; Burkart, Fehr, Efferson, & van Schaik, 2007; Douglas‐Hamilton, Bhalla, Wittemyer, & Vollrath, 2006; Fraser, Stahl, & Aureli, 2008; Koski & Sterck, 2009; Plotnik & de Waal, 2014). These species engage in prosocial behaviors, therefore, because they are inherently rewarding and not because they result in personal material gain (de Waal, 2007; de Waal et al., 2008; Horner, Carter, Suchak, & de Waal, 2011; Zaki & Mitchell, 2013).

In humans, social relationships are of primary importance for happiness and well‐being. Social connection is an integral component of human life, and interpersonal bonds are the source of numerous rewarding positive emotions (e.g., love and affection) that downregulate autonomic arousal and promote socioemotional attunement and concern (Fredrickson & Levenson, 1998; Goetz, Keltner, & Simon‐Thomas, 2010; Izuma, Saito, & Sadato, 2008; Oveis, Horberg, & Keltner, 2010). Empathy enables humans to share, understand, and respond to others’ emotional experiences through overlapping neural representations of self and others (Batson et al., 1997; Decety & Jackson, 2004; Zaki & Mitchell, 2013). Vicarious positive emotional experiences motivate acts of altruism and generosity even early in life and are seen in toddlers before the age of two (Aknin, Hamlin, & Dunn, 2012). Shared positive feelings may foster prosocial behaviors by activating neural systems (i.e., ventral striatum, anterior cingulate cortex, medial orbitofrontal cortex, thalamus, amygdala, and anterior insula) that support reward processing (Berridge & Kringelbach, 2015; Haber & Knutson, 2010). Prosocial behaviors including altruistic giving (e.g., spending money on others and giving to charities), empathic validation, emotional support, and cooperation activate reward network hubs such as the ventral striatum, among others (Declerck, Boone, & Emonds, 2013; Dunn, Aknin, & Norton, 2008; Harbaugh, Mayr, & Burghart, 2007; Inagaki & Eisenberger, 2012; Izuma, Saito, & Sadato, 2010; Morelli, Torre, & Eisenberger, 2014), that support reward processing as well as emotion and empathy more broadly (Cloutier, Heatherton, Whalen, & Kelley, 2008; Decety & Jackson, 2004; Mobbs et al., 2009; Morelli, Sacchet, & Zaki, 2015; Sescousse, Caldu, Segura, & Dreher, 2013).

Behavioral variant frontotemporal dementia (bvFTD) is a neurodegenerative disease that is characterized by a gradual deterioration of social behavior and empathy (Baez et al., 2014; Clark et al., 2015; Gleichgerrcht, Torralva, Roca, Pose, & Manes, 2011; Hsieh, Hornberger, Piguet, & Hodges, 2012). In bvFTD, atrophy in emotion‐ and reward‐relevant neural systems may alter patients’ responsivity to affective cues, thereby impairing their ability to share, intuit, and respond to the needs of others (Henry, Phillips, & von Hippel, 2014; Rankin et al., 2006; Snowden et al., 2008). Early and predominant atrophy in the anterior insula and anterior cingulate cortex diminishes emotional responsivity in certain contexts (Day et al., 2013; Kumfor & Piguet, 2012; Rosen & Levenson, 2009; Seeley et al., 2008; Sturm, Sollberger, et al., 2013). Laboratory studies have found that patients with bvFTD have dramatic deficits in social emotions (i.e., embarrassment) that promote prosocial behaviors (e.g., apologizing) and foster interpersonal relationships (Keltner & Buswell, 1997; Moll et al., 2011; Sturm, Allison, Rosen, Miller, & Levenson, 2006; Sturm, Ascher, Miller, & Levenson, 2008). Patients not only lose empathy and social decorum but also exhibit decline in affiliative personality traits (e.g., warmth) that are the foundation of enduring close relationships (Chiong et al., 2013; Rankin, Kramer, & Miller, 2005; Sollberger et al., 2009). These studies suggest that social relationships have reduced reward value in bvFTD and that patients are less motivated to partake in selfless acts that prioritize the feelings and needs of others. Although alterations in reward‐seeking (e.g., changes in eating, drug and alcohol use, and sexual behavior) are common in bvFTD and are associated with atrophy in reward network structures including the ventral striatum (Ahmed et al., 2015; Bocchetta et al., 2015; Perry & Kramer, 2015; Perry et al., 2014), whether alterations in reward processing also underlie patients’ waning social engagement is not well understood.

In this study we designed a novel computer‐based task (the “Giving Game”) to quantify prosocial behavior in patients with dementia. Although previous studies have determined that patients with bvFTD lack empathy, perspective‐taking, insight, and social understanding (Clark et al., 2015; Kumfor & Piguet, 2012; Rosen et al., 2014; Shany‐Ur et al., 2014; Snowden et al., 2008), relatively little is known about how prosociality deteriorates in bvFTD. Recent studies have shown that despite intact perceptions of fairness and basic bargaining skills, patients with bvFTD are unable to use socially relevant contextual information and, therefore, tend to make decisions that benefit themselves over others (Ibanez et al., 2016, 2017; Melloni et al., 2016; O'Callaghan & Hornberger, 2017; O'Callaghan et al., 2016).

We created a new paradigm (the “Giving Game”) that was modeled after simple token‐based tasks that have been used in non‐human primates to measure prosocial giving (de Waal et al., 2008; Horner et al., 2011). Participants played the Giving Game with the experimenter and on each trial chose how much money to give to themselves and to the experimenter. Prosocial giving was defined as the amount of money that participants gave to the experimenter when giving came at no cost to them and did not decrease their own winnings. Because shared feelings of reward motivate generosity and prosocial behavior (Morelli et al., 2015; Zaki & Mitchell, 2013), we hypothesized that prosocial giving would be compromised in bvFTD compared to healthy controls and patients with Alzheimer's disease (AD), a neurodegenerative disease that spares, and in some cases enhances, emotion‐relevant neural network connectivity and certain forms of socioemotional sensitivity (Goodkind et al., 2015; Sturm, Yokoyama, et al., 2013; Zhou et al., 2010). Furthermore, we hypothesized that atrophy in brain structures that support reward processing would be associated with alterations in prosocial giving.

## 2 MATERIALS AND METHODS

### 2.1 Participants

Seventy‐four participants were included in this study: 20 patients with bvFTD (Rascovsky et al., 2011), 15 with AD (McKhann et al., 1984), and 39 healthy controls. Participants underwent a multidisciplinary team evaluation at the University of California, San Francisco Memory and Aging Center that included a clinical interview, neurological exam, functional assessment, and neuropsychological testing (assessment of verbal and visual episodic memory, executive function, language, and visuospatial functioning). Functional assessments of dementia severity were obtained using the Clinical Dementia Rating Scale (CDR; Morris, 1993). The CDR Total (scores range from 0 to 3) and Sum of the Boxes (CDR‐SB) scores (scores range from 0 to 18, with higher scores on both CDR measures indicating greater functional impairment) were computed for each participant, providing indices of disease severity. The healthy controls were recruited from advertisements; underwent an identical neurological, cognitive, and imaging work‐up as the patients; and were free of current or previous neurological or psychiatric disorders. The study was approved by the Committee on Human Research at the University of California, San Francisco and all participants, or their surrogates, gave their informed consent. Table 1 presents the demographic, cognitive, and functional data for each group.

**Table 1:** Subjects characteristics classified by diagnostic group

	bvFTD	AD	Healthy controls
*n*	20	15	39
Age	63.9 (6.7)^a^	65.1 (10.2)	70.0 (5.0)
Sex (males: females)	12:8	6:9	14:25
Education	16.6 (3.3)	16.7 (4.7)	17.9 (2.0)
CDR total	1.5 (0.6)^a^	1.0 (0.4)^a^	0.0 (0.0)
CDR‐SB	8.1 (3.2)^a^	5.4 (2.3)^a^	0.0 (0.1)
MMSE	23.6 (5.5)^a^	22.9 (4.1)^a^	29.4 (0.9)
California verbal learning test short form 10‐min recall (/9)	4.7 (3.0)	1.9 (2.3)	b
Benson figure copy 10‐min recall (/17)	9.1 (4.6)	3.7 (4.7)^a^	11.9 (2.7)
Modified trails (correct lines per minute)	11.6 (53.3)^a^	9.9 (11.5)^a^	40.7 (14.0)
Modified trails errors	0.6 (1.2)	1.1 (1.6)^a^	0.2 (0.7)
Phonemic fluency (# correct in 60 s)	8.6 (4.8)^a^	11.9 (6.8)^a^	16.3 (4.8)
Semantic fluency (# correct in 60 s)	11.7 (5.5)^a^	11.5 (8.4)^a^	23.4 (7.7)
Design fluency correct (# correct in 60 s)	6.3 (4.5)^a^	5.8 (2.9)^a^	11.2 (4.7)
Design fluency repetitions	4.8 (4.2)^a^	1.4 (3.2)	1.3 (2.7)
Digits forward	5.6 (1.3)	4.9 (2.7)	6.6 (3.0)
Digits backward	3.4 (0.8)^a^	3.7 (1.3)	5.4 (2.8)
Benson figure copy (/17)	14.8 (1.7)	11.8 (5.5)^a^	15.3 (0.9)
Calculations (/5)	3.9 (1.0)^a^	3.3 (1.4)^a^	4.9 (0.3)
Boston naming test spontaneous correct (/15)	13.1 (2.7)	11.4 (2.5)	13.4 (4.8)
Peabody picture vocabulary test (/16)	14.8 (2.0)	14.6 (1.6)^a^	15.8 (0.5)
Stroop color naming (# correct in 60 s)	56.3 (24.0)^a^	50.7 (26.3)^a^	88.1 (15.1)
Stroop inhibition (# correct in 60 s)	28.2 (18.9)^a^	22.6 (17.5)^a^	52.5 (10.9)

bvFTD, behavioral variant frontotemporal dementia; AD, Alzheimer's disease; MMSE, Mini‐Mental State Examination; CDR Total, Clinical Dementia Rating Total score; CDR‐SB, Clinical Dementia Rating Sum of Boxes.

Means (*M*) and standard deviations (*SD*) are listed for each group unless otherwise noted.

a Tests in which the patients differed from the healthy controls in Bonferroni‐adjusted pairwise comparisons.

b The healthy controls received the California Verbal Learning Test‐II (16‐word list) instead of the Short‐Form. Their performance on the 20‐min delay was also in the average range (*M *=* *13.5, *SD *= 1.8).

### 2.2 Novel prosocial task: the Giving Game

We designed the Giving Game, a novel computer‐based task, to assess prosocial behavior in patients with dementia. This simple task was based on token tasks that are used to study prosocial giving in nonhuman primates (de Waal et al., 2008; Proctor, Williamson, de Waal, & Brosnan, 2013). Although patients with bvFTD are impaired on reward‐based decision‐making tasks (Bertoux, de Souza, Zamith, Dubois, & Bourgeois‐Gironde, 2015; Perry, Sturm, Wood, Miller, & Kramer, 2015), these tasks assess maximization of personal gain rather than selfless acts of generosity that do not influence one's own winnings. In the animal studies, two monkeys (a subject and a partner) are seated side by side and separated by a transparent partition, a configuration that allows full visual and vocal contact between the monkeys but no physical contact. The subjects first learn to associate two colored tokens with different reward outcomes and then to choose between the two tokens on subsequent trials. The subjects learn that if they choose the “selfish” token, they alone receive a reward (e.g., a slice of apple), and when they choose the “prosocial” token, the subjects and their partners both receive the same reward. Monkeys typically choose the prosocial token, giving to their partners even when there is no overt benefit to themselves, which suggests that prosocial behavior is normative and inherently rewarding in highly social species (de Waal et al., 2008).

We designed the Giving Game to be a human analog of the giving tasks that have been developed for animal studies. Each participant (i.e., akin to the subject in animal studies) plays the Giving Game with the experimenter (i.e., akin to the partner in animal studies) and has the opportunity to win money and to give money to the experimenter. During the task, the participant is seated next to the experimenter and completes a total of 36 decision‐making trials. The trials were presented in a randomized order for each participant; participants could take as long as they needed to respond to each trial. Prior to the task, the experimenter was introduced to the participant, explained the task, and obtained informed consent (for the patients, often with the assistance of a surrogate). This brief, semi‐standardized interaction enabled the experimenter to establish rapport with the participant, which is important for patients with cognitive and behavioral impairment because it minimizes confusion and creates an interpersonal connection. At the beginning of the task, the first names of the participant and the experimenter were entered into E‐prime (version 2.0) in order to personalize the task for each study pair.

The experimenter explained the task with the following instructions, which were presented on the laptop screen and read aloud by the experimenter:

In this task, we will both have the chance to win money. You will be asked to make some decisions, and we will be playing for quarters. On each trial, you will decide whether [participant's name] wins money, [experimenter's name] wins money, or we both win money. Your decisions will determine how much money [participant's name] and [experimenter's name] will receive at the end of the task. There are no right or wrong answers. Lets look at an example. On each trial, you will choose between Card A and Card B. If you chose Card A, you would win 25 cents, and I would win nothing. If you chose Card B, we would both win 25 cents. Which card would you choose, A or B?

**Figure 1:**
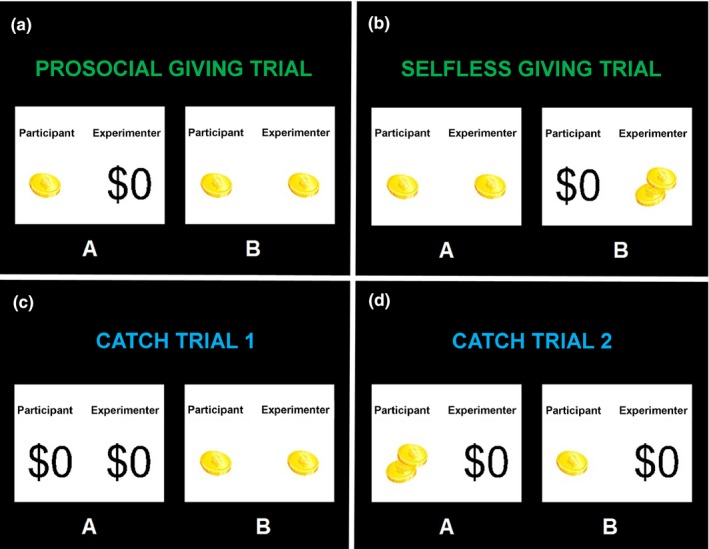
An example of each the four trial types: (a) *prosocial giving* (giving to the experimenter does not impact the participant's own winnings on that trial), (b) *selfless giving* (giving to the experimenter decreases the participant's winning on that trial), (c) *catch trials 1* (there is one choice that is advantageous to both the participant and the experimenter), and (d) *catch trials 2* (there is one choice that is advantageous to the participant without impacting the experimenter's winnings on that trial)

On each trial, the participant saw two cards, A and B, and was asked to choose between them (see Figure 1). The game includes two types of giving trials: on nine “*prosocial giving trials*,” participants can choose to give money to the experimenter or not (and their own winnings are the same regardless of whether they give), and on nine “*selfless giving trials*,” participants can choose to give money to the experimenter (but their winnings would decrease if they decide to do so). The game also includes two sets of catch trials in which one of the options is clearly advantageous for the participant; these trials were used as a validity check to determine whether the participant understood the task instructions and was paying attention throughout. On nine “*catch trials 1*,” there is one option that results in the participant and experimenter both winning more money than the other option, and on nine “*catch trials 2*,” there is one option where the participant wins more money than the other option (the experimenter wins the same amount regardless of which option is chosen).

For each set of trials, we computed the total amount of money that participants gave to the experimenter as our measures of giving. The total possible money that the participants could give to the experimenter in both the prosocial and selfless giving trials was $3.75. The total they could give to the experimenter in catch trials 1 was $3.50, and the total they could give in catch trials 2 was $2.25. For the prosocial and selfless giving trials, we also computed the total number of prosocial choices that participants made by summing the number of trials in which they made the prosocial choice. For catch trials 1 and 2, we computed the total number of “correct” choices participants made by summing the number of trials in which they chose the card in which the participant and the experimenter both received more money.

At the end of the game, a subset of participants (42 total: 15 patients with bvFTD, 11 patients with AD, and 16 controls) was asked two questions as an additional validity check to ensure that they had understood and believed the task instructions. First, they responded yes or no as to whether they believed that the experimenter would also win money after the task. Second, they were asked to rate on a 1–5 scale (1 = not confident, 3 = somewhat confident, and 5 = very confident) how confident they were that the experimenter would also win money at the end of the task.

### 2.3 Questionnaire measure of informant‐reported trait‐level empathy

Informants completed the Interpersonal Reactivity Index (IRI) and rated participants on their current empathic behavior. Informant ratings of personality and behavior in patients with dementia have been demonstrated to be a reliable measure of functioning (Rankin et al., 2004, 2005). The IRI is a psychometrically robust, multidimensional measure that evaluates distinct components of empathy (Cliffordson, 2002; Davis, 1983). Informants rated participants on each item on a scale of 1 (does not describe participant well) to 5 (describes participant very well). The IRI includes four subscales that measure distinct facets of empathy: the Empathic Concern subscale, which assesses the degree to which a person feels warmth and compassion toward others (e.g., “When he/she sees someone being taken advantage of, he/she feels kind of protective toward them”); the Personal Distress subscale, which measures the degree to which individuals experience anxiety and discomfort when they are exposed to others’ negative emotions (e.g., “Being in a tense emotional situation scares him/her”); the Perspective‐Taking subscale, which measures the extent to which individuals can adopt another's point of view of (e.g., “He/she believes that there are two sides to every question and tries to look at them both”); and the Fantasy Scale, which assesses the tendency to identify strongly with fictitious characters in books, movies, or plays (e.g., “When he/she watches a good movie, he/she can very easily put him/herself in the place of a leading character”). Whereas the Empathic Concern and Personal Distress subscales measure emotional empathy (i.e., the vicarious experience of others’ feelings via automatic affect‐sharing mechanisms), the Perspective‐Taking and Fantasy subscales measure cognitive empathy (i.e., comprehension of others’ emotions via emotion recognition and perspective‐taking). Subscale scores range from 7 to 35, with higher scores reflecting greater empathy.

### 2.4 Structural neuroimaging

The majority of participants (58 total: 17 bvFTD, 13 AD, 28 healthy controls) underwent research‐quality 3T structural magnetic resonance imaging (MRI). Patients were scanned within 4 months of the behavioral assessment, and healthy controls were scanned within 12 months. Images were obtained on a 3.0 Tesla Siemens (Siemens, Iselin, NJ, USA) TIM Trio scanner equipped with a 12‐channel head coil located at the UCSF Neuroscience Imaging Center. Whole brain images were acquired using volumetric MPRAGE (160 sagittal slices; slice thickness = 1.0 mm; field of view (FOV) = 256 × 230 mm^2^; matrix 256 × 230; voxel size 1.0 × 1.0 × 1.0 mm^3^; TR = 2,300 ms; TE = 2.98 ms; flip angle = 9°).

Structural T1 images were visually inspected for movement artifact, corrected for bias field, segmented into gray matter, white matter, and cerebrospinal fluid, and spatially normalized to Montreal Neurological Institute (MNI) space using Statistical Parametric Mapping (SPM) 12 (Friston, Ashburner, Kiebel, Nichols, & Penny, 2007). In all preprocessing steps, SPM12 default parameters were utilized with the exception of using the light clean‐up procedure in the morphological filtering step. Default tissue probability priors (voxel size: 2.0 × 2.0 × 2.0 mm^3^) of the International Consortium for Brain Mapping were used. Segmented images were visually inspected for adequate gray‐white segmentation. Gray matter maps were then smoothed with an 8 mm full‐width at half‐maximum Gaussian kernel. One patient with bvFTD was excluded for motion.

First, we conducted whole‐brain voxel‐based morphometry analyses to examine the brain atrophy patterns in bvFTD and AD compared with healthy controls (controlling for age, sex, and total intracranial volume). Next, we correlated prosocial giving with gray matter structural maps across all participants. We included age, sex, CDR Total, diagnosis (two variables dummy coded 1 and 0 for the three diagnostic categories), and total intracranial volume (a total of gray matter, white matter, and cerebrospinal fluid volume, to account for individual differences in head size) as nuisance covariates. In order to constrain the scope of the neuroimaging analyses and to offset the loss of power incurred by multiple comparison correction, we masked our analyses to structures in the reward network: thalamus, insula, anterior cingulate cortex, caudate, putamen, pallidum, amygdala, midbrain, and ventromedial prefrontal cortex (Perry & Kramer, 2015; Perry et al., 2014). See Fig.S1. *A priori* significance was established at uncorrected *p*_raw_ < .001. One thousand permutation analyses using combined peak and extent thresholds were run to derive a study‐specific error distribution to determine the one‐tailed *T*‐threshold for multiple comparisons correction at *p*_FWE_ < .05 (Nichols & Holmes, 2002). Permutation analysis is a resampling approach to significance testing by which a test statistic is compared to the null distribution derived from the present study's dataset and thus is an accurate representation of Type 1 error at *p *<* *.05 across the entire brain (Kimberg, Coslett, & Schwartz, 2007). Images were overlaid with MRIcron (www.mccauslandcenter.sc.edu/mricro/mricron) on the Montreal Neurological Institute template brain. Finally, to ensure that no other structures were involved outside of the reward network mask, we removed the mask and conducted follow‐up whole‐brain analyses of prosocial giving.

## 3 RESULTS

### 3.1 Clinical and demographic variables

Analyses of variance confirmed that the groups did not significantly differ in their mean education, *F*(2, 71) = 1.6, *p *=* *.21. A chi‐square test of proportions across the three diagnostic groups revealed no differences in their proportions of men and women, χ^2^(2, *N = *74) = 3.2, *p *=* *.20. Although the proportions of men and women in the AD and healthy control groups was similar, χ^2^(1, *N = *54) = 0.8, *p *=* *.78, the proportions of men and women in the bvFTD and healthy control groups approached significance, χ^2^(1, *N = *59) = 3.1, *p *<* *.08. Thus, we also included sex as a covariate in our analyses. Because there was a main effect of diagnosis on age at the time of testing, *F*(2, 71) = 5.9, *p *<* *.01, we also included age as a covariate in our analyses. The patients with bvFTD and AD were in the mild to moderate stages of disease severity as indicated by their CDR and cognitive testing scores.

### 3.2 Giving Game

#### 3.2.1 Catch trials

We first examined whether the participants performed at expected levels on the two sets of catch trials. We excluded two patients with bvFTD and one healthy control for incorrect responses to more than 33% of catch trials 1; all participants responded correctly to 100% of catch trials 2. Thus, the large majority of participants in each group understood the task, remembered the instructions, and paid attention throughout. See Table 2 for the behavioral data.

**Table 2:** Behavioral data from the Giving Game

	bvFTD	AD	Healthy controls
Giving trials			
Total money given to experimenter, *M* (*SD*)			
Prosocial giving trials	$2.71 (0.60)^a^	$2.80 (0.47)	$3.05 (0.39)
Selfless giving trials	$1.57 (0.86)	$1.70 (0.63)	$1.40 (0.75)
Total giving trials	$4.28 (1.20)	$4.50 (0.90)	$4.45 (0.90)
Percentage prosocial choices, %			
Prosocial giving trials	69.8 (26.4)^a^	76.3 (20.9)	88.3 (15.3)
Selfless giving trials	25.3 (27.2)	31.1 (21.5)	19.9 (23.3)
Total giving trials	47.5 (24.6)	53.7 (16.5)	54.1 (15.2)
Catch trials			
Total money given to experimenter, *M* (*SD*)			
Catch trials 1	$3.56 (0.33)	$3.60 (0.25)	$3.74 (0.04)
Catch trials 2	$2.25 (0.00)	$2.25 (0.00)	$2.25 (0.00)
Total catch trials	$5.81 (0.33)	$5.85 (0.25)	$5.99 (0.04)
Percentage correct trials, %			
Catch trials 1	93.2 (10.9)	94.8 (8.3)	99.9 (0.9)
Catch trials 2	96.3 (9.3)	91.1 (15.3)	97.4 (4.8)
Total catch trials	94.8 (8.0)	93.0 (10.4)	98.6 (2.4)
Confidence ratings			
Percentage who believed the experimenter would win money	93.3	90.9	75.0
Confidence rating that the experimenter would win money	4.7 (0.6)	3.6 (1.3)	3.4 (1.4)

Means (*M*) and standard deviations (*SD*) for total money given to the experimenter in the giving and catch trials. The total possible money that participants could give to the experimenter in both the prosocial and selfless giving trials was $3.75, and the total possible money that they could give to the experimenter in catch trials 1 was $3.50 and in catch trials 2 was $2.25. Performance rates on the catch trials are also presented. The confidence ratings were obtained using a 1–5 scale (1 = not confident, 3 = somewhat confident, and 5 = very confident) served as an additional validity check to assure that participants believed that the experimenter would win money at the end of the game.

a Signifies this value was different from healthy controls at *p *<* *.05.

#### 3.2.2 Giving trials

Analyses of covariance (controlling for age) revealed a main effect of diagnosis on prosocial giving trials, *F*(2, 66) = 4.1, *p *<* *.05, $\eta_{p}^{2}$ = .11. Bonferroni‐corrected pairwise comparisons revealed that patients with bvFTD gave less money to the experimenter than the healthy controls, *p* < .05. The patients with AD gave at an intermediate amount—more than the patients with bvFTD but less than the healthy controls—but did not differ significantly from either group (AD vs. bvFTD: *p* = 1.0; AD vs. healthy controls: *p* = .22). There was no main effect of diagnosis on selfless giving trials, *F*(2, 66) = 0.3, *p* = .72, $\eta_{p}^{2}$ = .01. Patients with AD gave the greatest amount, but this difference did not reach significance. A similar pattern was found when we examined each group's percentage of prosocial choices. There was a main effect of diagnosis on the percentage of prosocial choices made on the prosocial giving trials, *F*(2, 66) = 6.8, *p *<* *.01, $\eta_{p}^{2}$ = .16, with Bonferroni‐corrected pair‐wise comparisons revealing that patients with bvFTD made fewer prosocial choices than healthy controls (see Table 2). Patients with AD had the highest percentage of prosocial choices, but this difference did not reach significance. A one‐tail correlation analysis found that prosocial giving and selfless giving were modestly correlated across the entire sample, *r*(71) = .20, *p *<* *.05.

#### 3.2.3 Confidence ratings

The majority of participants in each group believed that the experimenter would win money after the task (see Table 2), and the groups did not differ in the proportion of participants who believed that the experimenter would receive money*,* χ^2^(2, *N = *42) = 2.5, *p *=* *.29. When we removed participants who reported that they did not believe the experimenter would win money, the main effect of diagnosis on prosocial giving remained significant, *F*(2, 31) = 4.9, *p *<* *.05, $\eta_{p}^{2}$ = .24. In addition, there was a significant difference among the groups in their confidence that the experimenter would win money at the end of the game, *F*(2, 37) = 4.6, *p *<* *.05. Bonferroni‐adjusted pairwise comparisons showed that the patients with bvFTD were significantly more confident than the healthy controls that the experimenter would win money, *p *<* *.05. The AD group's confidence rating fell between the other groups but did not significantly differ from the bvFTD (*p *<* *.07) or healthy control (*p *=* *1.0) groups. When we controlled for the confidence ratings, the main effect of diagnosis on prosocial giving held and even became stronger, *F*(2, 36) = 6.0, *p *<* *.01, $\eta_{p}^{2}$ = .25.

#### 3.2.4 Prosocial giving relates to informant‐reported empathic behavior

We ran multiple regressions to examine whether prosocial giving was associated with informant‐reported empathy as measured by the IRI. In step one of each regression, we entered age, sex, and diagnosis, and in step two we entered one of the IRI subscale totals. Across all participants, lower prosocial giving was associated with lower IRI Fantasy Scale scores (β = .25, *R* ^*2*^ *Change *= 0.06, *p *<* *.05). Prosocial giving was not related to scores on the Perspective‐Taking (β = −.07, *R* ^*2*^ *Change *= 0.01, *p *=* *.58), Personal Distress (β = −.01, *R* ^*2*^ *Change *= 0.00, *p *=* *.94), or Empathic Concern (β = .01, *R* ^*2*^ *Change* = 0.00, *p *=* *.95) subscales. Selfless giving, in contrast, was not associated with any of the IRI subscales: Fantasy Scale (β = .08, *R* ^*2*^ *Change* = 0.01, *p *=* *.48), Perspective‐Taking (β = .05, *R* ^*2*^ *Change *= 0.00, *p *=* *.71), Personal Distress (β = .12, *R* ^*2*^ *Change *= 0.01, *p *=* *.34), or Empathic Concern (β = .07, *R* ^*2*^ *Change *= 0.01, *p *=* *.61).

#### 3.2.5 Neural correlates of prosocial giving

**Figure 2:**
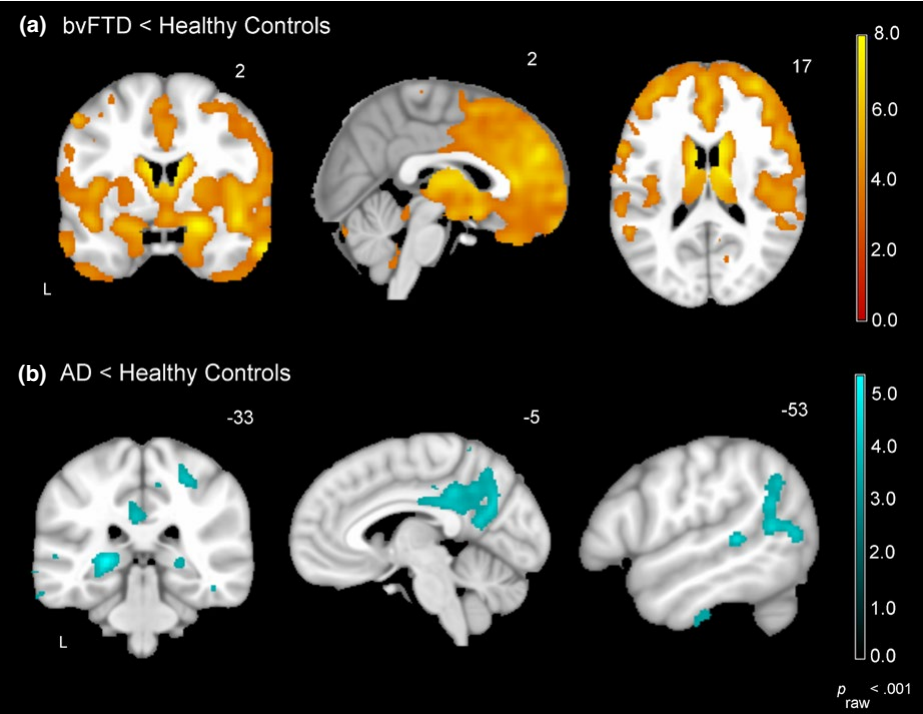
Atrophy patterns in the bvFTD and AD groups. Voxel‐based morphometry analyses (controlling for age, sex, and total intracranial volume) confirmed that each of the diagnostic groups had atrophy patterns that were consistent with their clinical syndrome (*p*_raw_ < .001). Compared to the healthy controls (*N* = 28), (a) patients with bvFTD (*N* = 17) had atrophy in the anterior insula, anterior cingulate cortex, amygdala, thalamus, prefrontal cortex, and orbitofrontal cortex whereas (b) patients with AD (*N* = 13) had atrophy in posterior cingulate cortex, precuneus, hippocampus, and lateral temporoparietal cortex

Voxel‐based morphometry analyses confirmed that the bvFTD and AD groups had atrophy patterns that were consistent with their clinical syndromes (see Figure 2). Patients with bvFTD had atrophy in the anterior insula, anterior cingulate cortex, amygdala, thalamus, prefrontal cortex, and orbitofrontal cortex whereas patients with AD had atrophy in posterior cingulate cortex, precuneus, hippocampus, and lateral temporoparietal cortex.

**Figure 3:**
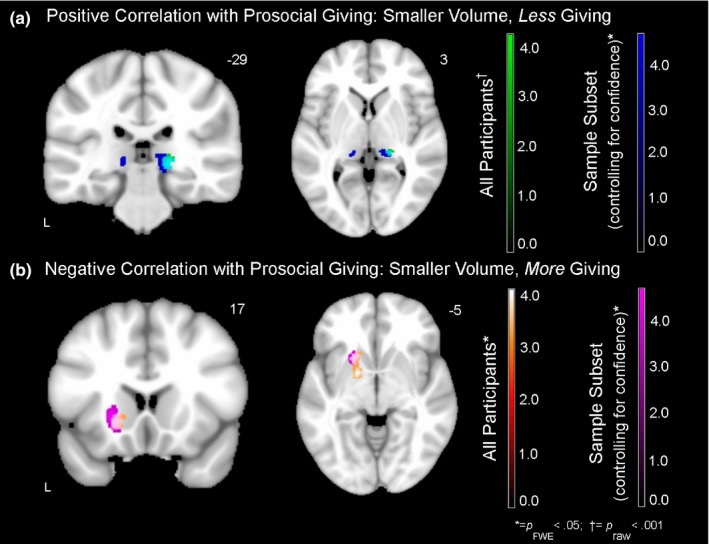
Neural correlates of prosocial giving. Regions in which smaller gray matter volume was associated with lower prosocial giving (cool colors) and greater prosocial giving (warm colors) at *p*_raw_ < .001 (covariates included age, sex, CDR Total, diagnosis, and total intracranial volume). (a) Across all participants (*N* = 58), smaller volume in the right pulvinar nucleus of the thalamus was associated with lower prosocial giving. In a subset of participants (*N* = 35), when we included participants’ confidence ratings as an additional covariate, smaller volume in the left pulvinar nucleus of the thalamus was also associated with lower prosocial giving. (b) In the analyses of both the entire sample and the subset of participants with confidence ratings, smaller volume in left ventral striatum was associated with greater prosocial giving

Across all participants, smaller volume in the right pulvinar nucleus of the thalamus was associated with lower prosocial giving (*p*_raw_ < .001 and *p*_FWE_ < .07). When we repeated this analysis and also controlled for participants’ confidence ratings in the task, these results held and became even stronger (see Table 3 and Figure 3). In addition, the left pulvinar nucleus also emerged as having a significant association with prosocial giving. Smaller volume in left caudate, left putamen, and left pallidum, however, was associated with greater prosocial giving (*p*_FWE_ < .05). These results were consistent, and became even stronger, when we added the confidence ratings as an additional covariate to this analysis.

**Table 3:** Anatomical correlates of prosocial giving

Anatomical region	Cluster volume (mm^3^)	*x*	*y*	*z*	Maximum *T*‐score
All participants					
Positive correlation with prosocial giving					
Right pulvinar	324^a^	20	−30	0	4.40
Negative correlation with prosocial giving					
Left pallidum	959^b^	−21	17	−2	4.63
Left caudate	c ^,^ b				
Left putamen	c ^,^ b				
Left caudate	338^b^	−17	3	−5	3.55
Sample subset (confidence ratings included as additional covariate)					
Positive correlation with prosocial giving					
Right pulvinar	611^b^	18	−30	−2	4.79
Right hippocampus	c ^,^ b	18	−28	−5	
Left pulvinar	186	−14	−30	2	3.89
Right orbitofrontal gyrus	10	24	35	−8	3.62
Negative correlation with prosocial giving					
Left pallidum	959^b^	−21	17	−2	4.63
Left caudate	c ^,^ b				
Left putamen	c ^,^ b				
Left pallidum	34	−17	3	−5	3.55

Across the entire sample (*N* = 58), volume loss in left ventral striatum was associated with greater prosocial giving, whereas volume loss in the right pulvinar nucleus of the thalamus was associated with lower prosocial giving when controlling for age, CDR Total, diagnosis, and total intracranial volume. In a subset of participants (*N* = 35), when we also included confidence ratings as an additional covariate in our analyses, our results were consistent with the analysis in the full cohort and were even more robust. MNI coordinates (*x*,* y*,* z*) are given for the maximum *T*‐score for the cluster. Statistical maps are superimposed on the MNI template brain. Results are significant at *p*_raw_ < .001.

a Denotes the cluster significant at *p*_FWE_ < .07.

b The cluster significant at *p*_FWE_ < .05.

c Signifies that these regions were included in the cluster above.

When we removed the reward network mask and ran a whole‐brain positive correlation analysis with prosocial giving, additional clusters (*p*_raw_ < .001) emerged in the left cerebellum (−5, −65, −35; cluster size = 289 mm^3^, max *T *=* *3.96), left fusiform gyrus (−27, −75, −8; cluster size = 250 mm^3^, max *T *=* *4.01), left paracentral lobule (−9, −23, 54; cluster size = 125 mm^3^, max *T *=* *3.93), and right inferior occipital lobe (41, −66, −12; cluster size = 68 mm^3^, max *T *=* *3.63). No additional clusters were found in the whole‐brain negative correlation analysis when the reward network mask was removed.

## 4 DISCUSSION

Human relationships are built on mutual feelings of interest, understanding, and caring (Batson, Duncan, Ackerman, Buckley, & Birch, 1981). Prosocial acts are motivated by shared positive feelings and depend upon the integrity of brain systems that support reward processing (Harbaugh et al., 2007; Marsh et al., 2014; Morelli et al., 2015). In bvFTD, patients become less engaged in the emotional lives of others and are less motivated to take care of others’ needs (Perry et al., 2001). Although it has been well documented that empathy and certain social emotions decline in bvFTD due to atrophy in emotion‐relevant neural circuits (Rankin et al., 2006; Sturm, Sollberger, et al., 2013; Zhou et al., 2010), whether bvFTD also impacts prosocial behavior has received less attention to date.

Using a novel computer‐based task, we found that patients with bvFTD had diminished prosocial giving compared to healthy controls. They gave less money to the experimenter even when it was at no expense to do so and despite being highly confident (even more confident than the healthy controls) that the experimenter would receive money at the end of the task. Patients with AD gave an intermediate amount that was in between that of the bvFTD and healthy control groups. Lower prosocial giving was associated with informant‐reported empathy deficits and atrophy in reward‐relevant circuits. Whereas lower prosocial giving was primarily associated with smaller gray matter volume in the right pulvinar nucleus of the thalamus, greater prosocial giving was associated with atrophy in the left ventral striatum. When we removed the mask, atrophy in several posterior regions (e.g., cerebellum, fusiform gyrus, and paracentral lobule) also emerged as being related to lower prosocial giving. Atrophy in these structures may also have interfered with participants’ ability to monitor social cues and to take actions that would benefit the experimenter.

Prosocial actions are inherently rewarding and are motivated by the pleasurable feelings that accompany helping another (Zaki & Mitchell, 2013). These shared positive emotions promote affiliative behaviors that strengthen social bonds between the giver and the benefactor by signaling concern and camaraderie (Batson et al., 1981; Decety & Jackson, 2004). Because empathy (i.e., shared emotional experiences as well as a cognitive understanding of others’ affective states) is critical for motivating compassionate actions in response to others’ needs, loss of empathy in bvFTD may render patients unresponsive to other people and less motivated to act in kind and generous ways (Moll et al., 2011; Rankin et al., 2005). In line with recent work on social decision‐making in bvFTD, our results suggest that patients prioritize their own needs and desires over those of others and may fail to integrate complex social information when making choices (Ibanez et al., 2016, 2017; Melloni et al., 2016; O'Callaghan & Hornberger, 2017; O'Callaghan et al., 2016). We also found that prosocial giving moderately correlated with one of the cognitive empathy subscales on the IRI (i.e., the Fantasy Scale), which suggests that although prosocial actions may depend in part on empathy, our measure of prosocial giving captures a distinct, yet related construct. The Fantasy Scale assesses the degree to which an individual identifies with characters in books, movies, or plays, and it is possible that this ability facilitated participants’ engagement in the Giving Game and inspired feelings of generosity.

In bvFTD, neurodegeneration of predominantly right hemisphere emotion‐relevant neural systems has been associated with deficits in emotion recognition, perspective‐taking, and empathy (Baez et al., 2016; Eslinger, Moore, Anderson, & Grossman, 2011; Ibanez & Manes, 2012; Kumfor et al., 2014; Shany‐Ur et al., 2012), abilities that foster social relationships and feelings of interpersonal connection. We found that atrophy in the right pulvinar nucleus of the thalamus (and left, when we accounted for confidence ratings) was associated with lower prosocial giving. The strongest thalamic cluster was centered in the lateral portion of the right medial pulvinar nucleus (Krauth et al., 2010), an area that has strong projections to the insula and anterior cingulate cortex (Benarroch, 2015; Romanski, Giguere, Bates, & Goldman‐Rakic, 1997) and is integral for relaying incoming sensory information to distributed neural systems that support emotion and reward processing. In bvFTD, breakdown in this afferent system may interfere with generosity by hampering patients’ access to internal emotional cues that typically motivate prosocial behaviors. Recent findings suggest that in at least one subtype of bvFTD, those with mutations in the *C9ORF72* gene, the pulvinar is an early site for neurodegeneration (Lee et al., 2014), an atrophy pattern that may help to distinguish this group from other forms of frontotemporal dementia (Bocchetta et al., 2016; Whitwell et al., 2015).

The ventral striatum is a key hub in reward circuitry that activates during the anticipation and receipt of numerous types of rewards. Money, pleasant odors, attractive people, smiling faces, and appetitive cues all recruit the ventral striatum (Kuhn & Gallinat, 2012; Sescousse et al., 2013). During vicarious reward, despite the fact that observers do not directly receive rewards themselves, the ventral striatum is also active, which perhaps reflects its role in triggering the mutual positive feelings that arise from prosocial actions (Mobbs et al., 2009; Morelli et al., 2015). In bvFTD, atrophy in the right ventral striatum is common and has been associated with increased seeking of primary rewards (e.g., food and alcohol), suggesting that patients with bvFTD may be hyper‐reactive to certain types of nonsocial rewarding cues (Perry et al., 2014). Predominantly right‐sided atrophy in bvFTD has also been associated with abnormal responding to musical sounds, another type of nonsocial cue that carries inherent reward value (Fletcher et al., 2015). Left‐lateralized damage, in contrast, has been linked to enhancements in positive socioemotional traits that are important for interpersonal functioning. Left ventral striatal damage has been linked to higher levels of positive emotion (Sturm et al., 2014) and even excessive generosity (Ferreira‐Garcia, Fontenelle, Moll, & de Oliveira‐Souza, 2014). Atrophy in left orbitofrontal cortex has been associated with increased agreeableness, a personality trait that fosters interpersonal connection through positive emotion and warmth (Rankin et al., 2004). Left‐hemisphere fronto‐striatal dysfunction, by loosening the regulation of positive affect, may heighten prosocial tendencies in some cases. Taken together, these findings raise the interesting possibility that right and left striatal damage may have differential effects on social and nonsocial reward processing. Whereas right striatal damage may predominantly alter patients’ responsivity to nonsocial rewards, left striatal damage may more notably influence their responsivity to social rewards. Whether patients exhibit increased or decreased reward sensitivity in social or nonsocial contexts may reflect the exact ways in which lateralized striatal microcircuits are dysfunctional.

This study has limitations to consider. First, we found an association between prosocial giving and the Fantasy Scale of the IRI, but no other associations emerged between prosocial giving and the other IRI empathy subscales. Empathy is a complex construct that refers to the ability to know (i.e., cognitive empathy) and to feel (i.e., emotional empathy) others’ affective states (Decety & Jackson, 2004). The Fantasy Scale measures one facet of cognitive empathy, the ability to identify others’ feelings via appraisal processes (Davis, 1983). We did not find an association between prosocial giving and the IRI emotional empathy subscales. Emotional empathy refers to the ability to simulate another's affective state via physiological and behavioral mirroring systems, a process that also fosters the vicarious experience of reward (Decety & Sommerville, 2003). The IRI, which measures trait‐level empathy and focuses on empathy for negative emotions, may therefore have a limited association with participants’ positive empathy and prosocial behavior in this context (Ickes, 2009). It is also possible that participants were motivated to give during this task not only because of vicarious reward but also because of cognitive empathy and understanding of social rules (e.g., knowledge of social norms, adherence to task demands, etc.). As in the animal studies, we measured prosocial behavior as a downstream product of vicarious reward experience but did not explicitly query participants about their subjective emotional experience. Future studies could investigate patients’ self‐reported affect in response to rewarding cues and prosociality. Second, we examined prosocial behavior during a task in which participants chose to give to an experimenter who was a neutral, yet friendly study experimenter. Previous studies have largely focused on prosocial behaviors in response to suffering rather than those that are motivated by the shared experience of reward. Laboratory studies, for example, have found that individuals who experience the suffering of others more intensely (as measured by greater physiological reactivity and subjective emotional experience) are more generous in their donations to charities that aim to alleviate the suffering than those who are less emotionally moved (Sze, Gyurak, Goodkind, & Levenson, 2012). Future studies that examine the neural correlates that support responding compassionately to another's suffering versus those engaged when sharing positive emotional experiences will be necessary to determine how negative and positive emotions may moderate prosocial actions. Third, the participants in this study were highly educated, which may limit the generalizability of our results. Although we designed the Giving Game to minimize cognitive load, it is possible that cognitive impairment in the patient groups impacted their performance. Deficits in working memory, attention, and calculations were evident in the patients and, therefore, may also have influenced their decisions and behavior. Fourth, the Giving Game was inspired by token‐based paradigms used in animal studies that measure simple acts of generosity. Highly social species (e.g., elephants, great apes, and dolphins) that exhibit prosocial behaviors such as giving, targeted helping, and consolation behaviors are also those that are capable of complex social‐cognitive acts such as mirror self‐recognition (Seeley & Sturm, 2006). These species are also united in that they have relatively large proportions of von Economo neurons, large neurons that are important for integrating socioemotional cues, which are also especially vulnerable in bvFTD (Seeley et al., 2006). Future work that relates the integrity of these specialized neurons to the degradation of prosociality in bvFTD will help to delineate the biology of these exceptional behaviors.

This study found evidence that prosocial behavior, as assessed by a novel computer‐based task of prosocial giving, is impaired in bvFTD compared to healthy controls. Although patients with AD gave an amount that was intermediate, falling between the bvFTD and healthy control groups, on the prosocial trials, they tended to give more than the other groups on the selfless giving trials, though this difference did not reach significance. Although patients with bvFTD reported being highly confident that the experimenter would receive the money they gave them at the end of the task, they gave less money even when it was at no expense to them. Lower prosocial giving was associated with deficits in informant‐reported empathic behavior as determined by informant‐report. Alterations in prosocial behavior were associated with distinct, lateralized patterns of brain atrophy. Whereas, smaller volume in the right pulvinar nucleus of the thalamus, a key region in reward processing and a hub that interacts with emotion‐relevant networks, was associated with lower prosocial giving, atrophy in left ventral striatum was associated with greater giving. These findings suggest that in bvFTD there is neurodegeneration of reward‐relevant neural systems that has specific effects on how patients value others and the extent to which they are motivated to share in others’ positive experiences. The study of prosocial behavior in neurodegenerative diseases has the potential to help to elucidate the anatomical basis of generosity in humans.

## CONFLICT OF INTEREST

None declared.
