# Supplementary figures and images for: Prosocial deficits in behavioral variant frontotemporal dementia relate to reward network atrophy
Source: Brain Behav. 2017 Sep 14;7(10):e00807. doi: 10.1002/brb3.807 (PMC5651391; doi:10.1002/brb3.807)

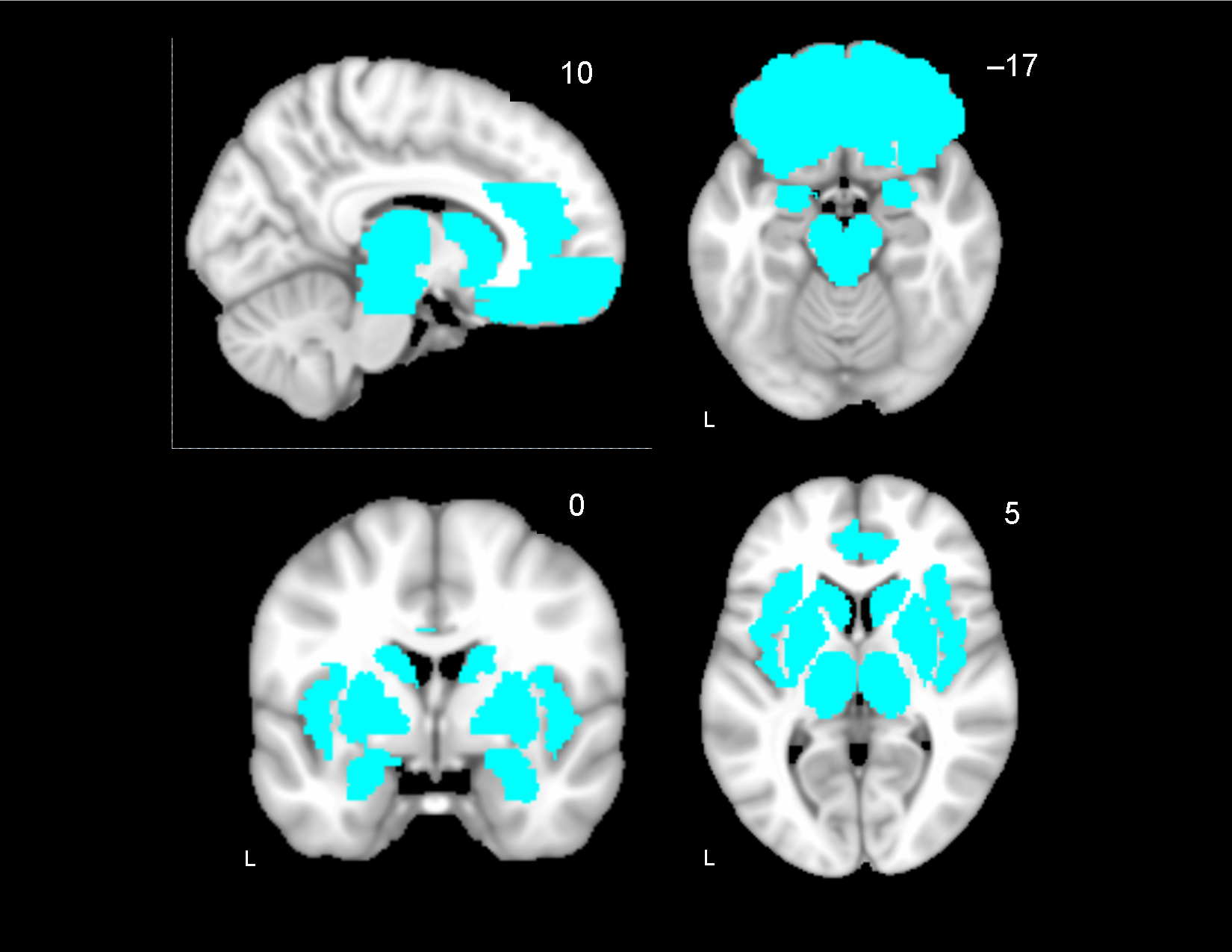

Supplement: Supplementary file 1 [file BRB3-7-e00807-s001.tif]
